# The influence of sublethal blue light exposure on human RPE cells

**Published:** 2009-09-21

**Authors:** Cora Roehlecke, Annette Schaller, Lilla Knels, Richard H.W. Funk

**Affiliations:** 1 Institute of Anatomy, TU Dresden, Dresden, Germany; 2 CRTD/DFG-Center for Regenerative Therapies Dresden – Cluster of Excellence, Dresden, Germany

## Abstract

**Purpose:**

To evaluate the in vitro response of retinal pigment epithelial (RPE) cells to a nonlethal dose of blue light.

**Methods:**

The human RPE cell line ARPE-19 was irradiated with blue light (405 nm) at an output power of 1 mW/cm^2^ or 0.3 mW/cm^2^. The following parameters were studied: metabolic activity; apoptosis; reactive oxygen species (ROS) production; mitochondrial membrane potential (MMP); ultrastructural changes of mitochondria; production of advanced glycation endproducts (AGEs); and stress-related cellular proteins.

**Results:**

Nonlethal doses of blue light irradiation significantly reduced ARPE-19 metabolic activity and MMP while increasing intracellular ROS levels and expression of stress-related proteins heme oxygenase-1 (HO-1), osteopontin, heat shock protein 27 (Hsp-27), manganese superoxide dismutase (SOD-Mn), and cathepsin D. Blue light irradiation also induced ultrastructural conformation changes in mitochondria, resulting in the appearance of giant mitochondria after 72 h. We further found enhanced formation of AGEs, particularly N^ε^-(carboxymethyl) lysine (CML) modifications, and a delay in the cell cycle.

**Conclusions:**

ARPE-19 cells avoid cell death and recover from blue light irradiation by activating a host of defense mechanisms while simultaneously triggering cellular stress responses that may be involved in RPE disease development. Continuous light exposure can therefore detrimentally affect metabolically stressed RPE cells. This may have implications for pathogenesis of age-related macular degeneration.

## Introduction

Age-related macular degeneration (AMD) is the leading cause of progressive blindness in elderly in developed countries [1]. In recent years, the number of people affected has steadily increased as more than 35% of the population is now over age 75 and showing disease symptoms [1]. The pathogenesis of AMD is poorly understood, and to date, there is no efficient cure or prevention. Several epidemiologic studies suggest that long-term history of exposure to light may trigger the onset of AMD [2-5].

The human retina is protected from high-energy ultraviolet light by the cornea and lens, which absorb ultraviolet (UV) light below 400 nm, but can be damaged by visible light [6]. Components of the visible spectrum can be absorbed by biologic chromophores in retinal pigment epithelial (RPE) cells, causing cellular dysfunction and even death of cells [7]. The blue region of the spectrum (400–500 nm) has relatively high energy and can penetrate through tissues to cells and their organelles. Blue light in particular is known to damage retinal tissue [8-12]. Much attention has been given to chromophores formed by rhodopsin intermediates in the photoreceptor outer segments, such as the protein A2E, a major component of lipofuscin [7,13]. These chromophores have been regarded as the major source of radicals in RPE cells, however, it has recently been shown that blue light can also damage lipofuscin-free RPE cells [14,15]. Cell culture studies revealed that blue light directly induces the production of reactive oxygen species (ROS) in RPE mitochondria [14] and leads to apoptosis [16], potentially triggered by ROS damage to mitochondrial DNA (mtDNA) [17].

In this study, we established an in vitro model system in which blue light irradiation of RPE cells induces mild stress without causing cell death. It is likely that the accumulation of subthreshold damage to cellular processes produces long-term subapoptotic cell stress that finally leads to the apoptosis associated with AMD. Thus, we applied subapoptotic doses of blue light to our RPE cell model and assayed indicators of subtle cellular change. We were particularly interested in effects on mitochondrial morphology and membrane potential, metabolic activity, stress-related protein levels, the cell cycle, and formation of advanced glycation endproducts (AGEs), particularly N^e^-(carboxymethyl) lysine-modified proteins. AGEs are a heterogenous group of reaction products formed by nonenzymatic Maillard reactions between a protein`s primary amino group and a carbohydrate-derived aldehyde group. Intracellular formation of AGEs is a crucial pathological process in various retinal degenerations, including AMD.

## Methods

### Cell culture

The human retinal pigment epithelial cell line ARPE-19 (ATCC, Rockville, MD) [18], was grown in a 1:1 mixture of Dulbecco’s modified eagle medium (DMEM) and Ham’s F12 (PAN Biotech, Aidenbach, Germany) supplemented with 10% fetal calf serum (Biochrom, Berlin, Germany). Cells were used at passages 25 to 30. They were counted with a Casy cell counter (Schärfe System, Reutlingen, Germany) before seeding.

### Exposure of ARPE-19 cells to blue light

Illumination was produced by a LED-based system generating 405 nm blue light at an output power of either 0,3 mW/cm^2^ or 1 mW/cm^2^. LED arrays were developed in cooperation with Hydrosun Medizintechnik GmbH, Müllheim, Germany. Cells were irradiated in six-well chambers (TPP, Trasadingen, Switzerland) or microslides (ibidi, Munich, Germany) for 3, 24, or 72 h.

### Chemicals and antibodies

The following chemicals were used in our experiments: propidium iodide, RNase, saponin, triton-X and tetramethylrhodamine isothiocyanate-labeled phalloidine (Sigma-Aldrich, St. Louis, MO); bovine serum albumin (BSA; Serva, Heidelberg, Germany); NP-40 (Roche AG, Basel, Switzerland), 2’7’-dihydro ethidium (DHE), 5-(and-6)-chloromethyl-2’,7’-dichlorodihydrofluorescein diactate acetyl ester (CM-H_2_DCFDA), and 5,5′,6,6’-tetrachloro-1,1’,3,3′-tetraethylbenzimidazol-carbocyanine iodide (JC-1; Molecular Probes, Leiden, The Netherlands). The following antibodies were used in our studies: 1:1,500 polyclonal rabbit anti-CML antiserum (gift of Erwin D. Schleicher, Department of Medicine IV, University of Tübingen, Tübingen, Germany) [19]; heme oxygenase-1 (HO-1) and heat shock protein 27 (Hsp-27; Stressgen,, Victoria, Canada); osteopontin (Vector Laboratories, Burlingame, CA); manganese superoxide dismutase (SOD-Mn; Calbiochem, Cambridge, MA); and cathepsin D (Bio Genex, San Ramon, CA). Secondary antibodies included fluorescein isothiocyanate (FITC)–conjugated anti-rabbitIgG, anti-goat IgG or anti-mouse IgG (Dianova, Hamburg, Germany) at a final dilution of 1:80.

### Detection of metabolic activity

Cell metabolic activity was quantitatively assessed using a resazurin assay according to the manufacturer’s instructions (Sigma-Aldrich) [20,21]. Cells were irradiated with blue light, and the assay was performed. Cell-free wells containing medium and resazurin were used as blanks.

### Measurement of intracellular ROS production

Cells were incubated with DHE or H_2_DCFDA at a final concentration of 10 μM for 30 min at 37 °C in the dark. Excess dye was removed by washing in PBS. Fluorescence intensity was measured in a FACSCalibur cytofluorimeter (Becton-Dickinson, Heidelberg, Germany). For each analysis, 10,000 cells were recorded.

### Detection of apoptosis

Apoptosis was quantified by flow cytometry to detect cells with a subdiploid DNA peak and by M30 Apoptosense ELISA (Peviva; Axxora, Lörrach, Germany). The assay for subdiploid DNA (sub-G_1_ peak) was performed as described by Nicoletti et al. [22]. Cells were fixed in cold 70% ethanol for 5 min. Next, 50 μl PBS (Pan Biotech GmbH, Aidenbach, Germany; components of Dulbecco's PBS: potassium chloride, potassium dihydrogen phosphate, sodium chloride, di-sodium hydrogen, pH 7.4) with 1% horse serum containing 1 μg propidium iodide and 10 μg RNase was added, and the mixture was incubated for 30 min at room temperature. Flow cytometry was performed using a FACSCalibur cytofluorimeter. The number of cells per histogram was 10,000. With this method, cells with a normal DNA peak were distinguishable from apoptotic cells. Necrotic cells were detected by incubating unfixed cells for 5 min with 0.01 mg/ml propidium iodide, and this was followed by cytofluorimetric analysis.

The M30-Apoptosense ELISA was performed as follows: cells were lysed by adding 100 µl 10% NP-40 per well. After 5 min incubation at room temperature, 25 µl of the lysate was transferred to each well of M30 coated microstrips. The samples were assayed in triplicate and the standards in duplicate. The extinctions were averaged before calculation of M30 values. Photometrical analysis at 450 nm was conducted using a TECAN Sunrise ELISA Reader (Tecan trading AG, Switzerland).

### Measurement of mitochondrial membrane potential

Mitochondrial membrane potential (MMP) was assessed by measuring the potential-dependent accumulation of JC-1 [23-25]. After treatment with blue light, cells were stained with JC-1 (1 mg/ml stock solution in DMSO diluted to 1 μg/ml with medium) for 10 min at 37 °C. Fluorescence intensity was imaged using a fluorescence microscope (IX81; Olympus, Hamburg, Germany) equipped with a dual view imaging system and an Olympus Megaview camera (statistical analysis of JC-1 labeled mitochondria in ARPE-19 cells from 40 images of 8 separate experiments with 5 replicates each).

### Electron microscopy

Prior to transmission electron microscopy, cells were fixed for 2 h at room temperature (RT) in 0.1 M sodium cacodylate buffer (pH 7.4) containing 2.5% glutaraldehyde. The samples were incubated in 1% OsO_4_ in 0.1 M cacodylate buffer for 2 h at RT. Afterwards, samples were contrasted with 3% uranyl acetate en bloc, dehydrated through a graded alcohol series at RT, and incubated for 2 h with 1:1 ethanol-epon. Thereafter samples were embedded in epon overnight and the flat embedding molds filled with epon were polymerized for 48 h at 60 °C. Ultrathin sections (65 nm) were mounted on grids and contrasted with uranyl acetate (8 min) and lead citrate (2 min). Ultrastructural studies were performed with an EM 906 electron microscope (Carl Zeiss, Oberkochen, Germany). All buffers, fixatives, and embedding materials for electron microscopy were purchased from Serva.

### Cytofluorimetric analysis of intracytoplasmic proteins

To detect N^ε^-(carboxymethyl) lysine (CML)-modified proteins and cytoplasmic proteins, we fixed cultured cells in 2% (w/v) formaldehyde in PBS for 20 min and centrifuged. The cells were resuspended in wash buffer, which contained 0.5% BSA in PBS, and permeabilized for 20 min using 0.5% (w/v) saponin in wash buffer. After incubation with primary antibodies for 30 min, the cells were washed twice in PBS containing 0.5% BSA and 0.5% (w/v) saponin. Then cells were incubated with a FITC-conjugated anti-rabbit IgG or anti-mouse IgG for 60 min at RT. Cells were washed in PBS, resuspended in 400 μl PBS, and analyzed by flow cytometry (FACSCalibur). At least 10,000 cells were analyzed.

### Cell cycle analysis

After cells were harvested, 5×10^5^ cells were prepared using the CycleTest^TM^ Plus DNA Reagent Kit (Becton Dickinson) and analyzed by flow cytometry. Approximately 20,000 events were recorded per analysis. Cell cycle phase distribution was analyzed by ModFit LTTM 2.0.1 (Verity Software House Inc., Topsham, ME).

### Statistical analysis

Values are given as mean±SD, with n equal to the number of independent experiments. Unless stated otherwise, all assays were performed at least five times. Statistical analysis was performed by one-way ANOVA with correction for post hoc multiple comparisons according to Bonferroni using SPSS, version 12.0 (SPSS, Chicago, IL). Significance was accepted at p<0.05. Histograms and images are examples of series from similar experiments.

## Results

### Viability and apoptosis of ARPE-19 cells

We used ARPE-19, a human adult diploid RPE cell line similar to RPE in vivo, to test the effects of sublethal exposure to blue light. Viability and cell number after exposure to blue light for 24, 48, and 72 h did not vary significantly in any of the experiments described here. Cell viability was assayed by trypan blue and propidium iodide exclusion, and microscopic and flow cytometric evaluations were performed. None of our samples showed a sub-G1-peak, indicative of apoptotic cells. Consistent with this, M30 Apoptosense ELISA detected no blue-light-induced apoptosis after 24, 48, or 72 h (data not shown). Thus, the blue-light exposure delivered in our setup is indeed sublethal for ARPE-19 cells.

### Metabolic activity of ARPE-19 cells

**Figure 1:**
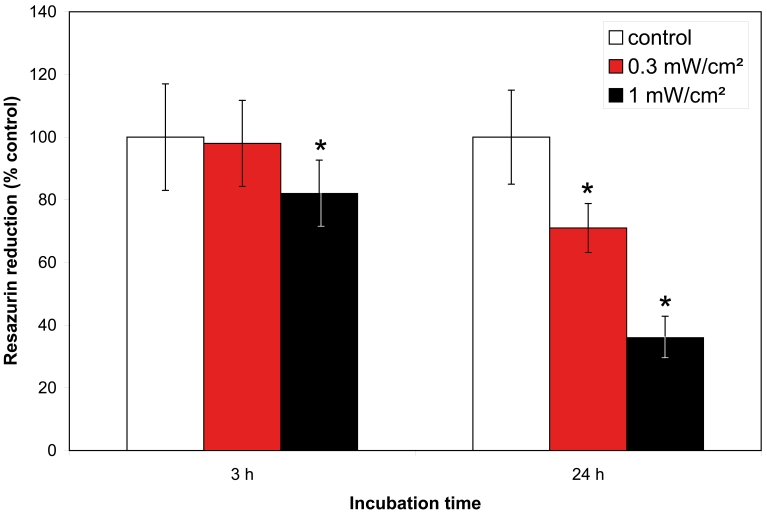
Effect of blue light on metabolic activity. Metabolic activity of ARPE-19 cells measured by spectrophotometric detection of resazurin. Cells were unirradiated (white bars) or irradiated with either 1 mW/cm^2^ (black bars) or 0.3 mW/cm^2^ (red bars) for 3 h and 24 h. Bars represent mean±SD from n=5 separate experiments; The asterisk (*) indicates a p<0.05 (one-way ANOVA and Bonferroni test).

To determine the impact of blue-light exposure, we quantified ARPE-19 cell injury by measurement of the reduction of resazurin. Metabolic activity of the cells was significantly decreased after irradiation for either 3 or 24 h with blue light at an output power of 1 mW/cm^2^. It was also significantly decreased after 24 h irradiation at an output power of 0.3 mW/cm^2^ (Figure 1).

### Evaluation of intracellular oxidative stress

**Figure 2:**
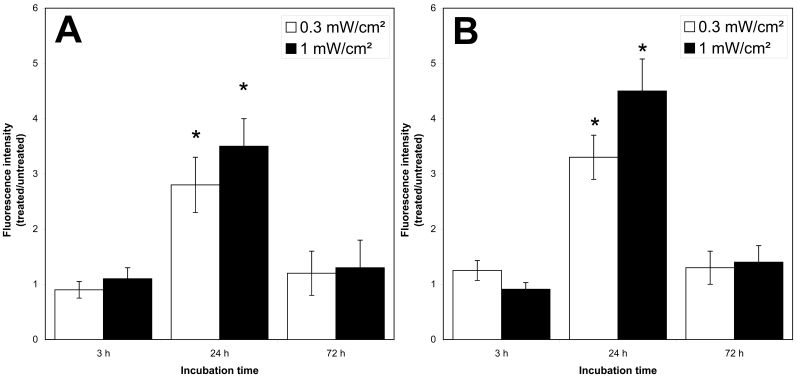
Effect of blue light on intracellular ROS. Flow cytometric analysis of intracellular ROS levels with the oxidant-sensitive dyes DHE (**A**) and CM-H2DCFDA (**B**) in ARPE-19 cells. Cells were irradiated with 1 mW/cm^2^ (dark bars) or 0.3 mW/cm^2^ (light bars) for 3, 24, or 72 h. Unirradiated cells were used as negative controls. The graph displays median fluorescence intensity ratios of irradiated cells versus unirradiated controls. Bars represent mean±SD from n=5 separate experiments; The asterisk (*) indicates a p<0.05 (one-way ANOVA and Bonferroni test).

Intracellular ROS level is an important biomarker for oxidative stress, with increased ROS levels generally indicating greater oxidative stress. To test for blue-light-induced ROS production in our system, we treated ARPE-19 cells with blue light for 3, 24, or 72 h. Intracellular ROS formation was measured by incubating cells with DHE or CM-H_2_DCFDA. The dyes, DHE and CM-H_2_DCFDA, have been used to measure intracellular generation of superoxide and hydrogen peroxide, respectively. We found that 24 h of exposure to blue light significantly stimulated ROS production as evidenced by oxidation of DHE and H_2_DCFDA (Figure 2). Moreover, this effect was dependent on the output power of the blue light.

### Evaluation of MMP

**Figure 3:**
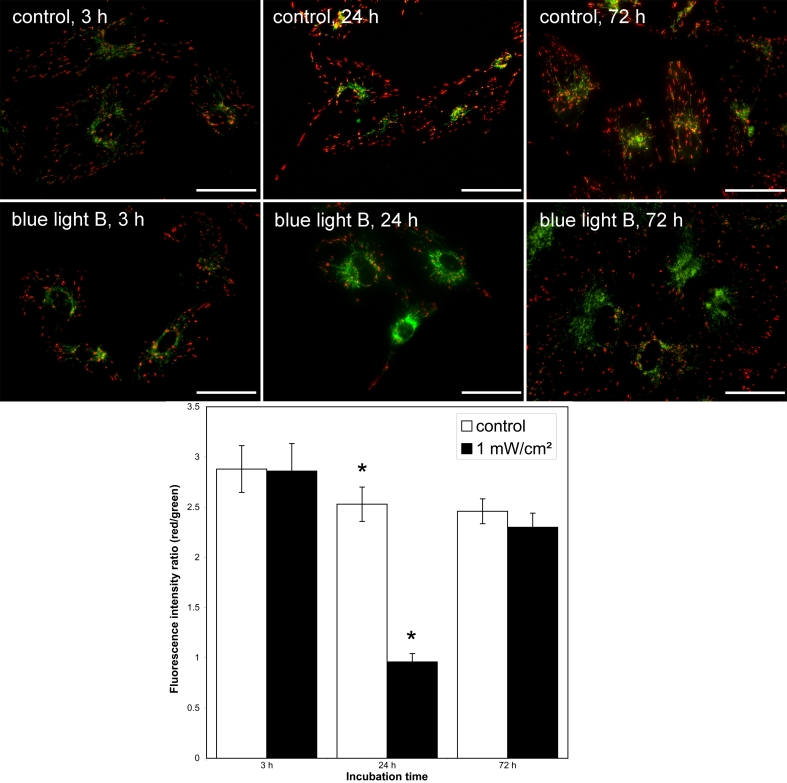
Effect of blue light on MMP. **A**: Representative images of JC-1 labeled mitochondria of ARPE-19 cells. Unirradiated cells (control) and irradiated cells (1 mW/cm^2^) were stained after 3 h, 24 h, or 72 h. Each panel is representative of 8 separate experiments (Bar=50 μm). **B**: Statistical analysis of JC-1 labeled mitochondria of ARPE-19 cells. Unirradiated cells (control) and irradiated cells (1 mW/cm^2^) were stained after 3 h, 24 h, or 72 h. 40 images from n=8 separate experiments were taken and red and green fluorescences of JC-1 were counted to quantify MMP. Blue light treatment for 24 h resulted in a significant decrease in red-green ratio of JC-1. The asterisk (*) indicates a p<0.05 (one-way ANOVA and Bonferroni test).

To evaluate the effect of blue light on MMP, we used fluorescence microscopy to detect the potential-dependent accumulation of JC-1 (Figure 3). After 3 h of irradiation, no significant effect on MMP was detectable compared to control cells. However, after 24 h, blue light irradiation decreased red JC-1 aggregate fluorescence and led to predominantly green JC-1 monomere fluorescence, indicating decreased MMP. Most mitochondria in the control cells displayed red fluorescence by JC-1 aggregates. Over time, in both the control and irradiated cells, intact mitochondria (red) redistributed to the cell periphery and depolarized mitochondria (green) located to the perinuclear region. This redistribution process was most prominent after 72 h of blue light irradiation. We further observed the elongation of several mitochondria. The average MMP of control and treated cells did not vary significantly after 72 h of irradiation.

### Electron microscopic observations of mitochondria

**Figure 4:**
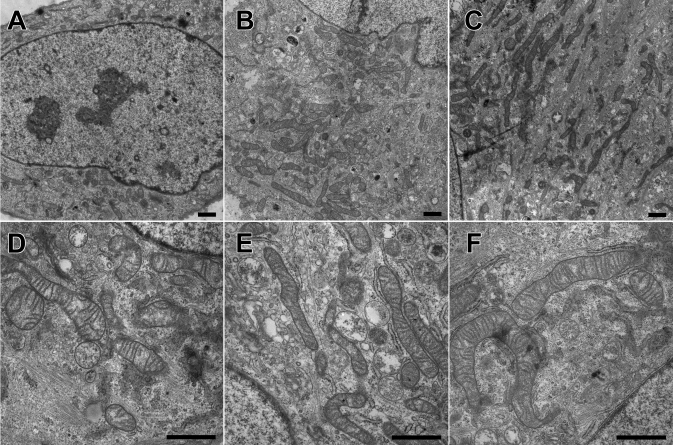
Effect of blue light on mitochondrial shape. The figure shows electron micrographs of sections from untreated ARPE-19 cells (**A, D**), irradiated cells with an output at 0.3 mW/cm^2^ (**B, E**) and 1 mW/cm^2^ (**C, F**) for 72 h (Bar=1 μm). Irradiated cells showed mitochondria with unusually elongated profiles.

The changes in mitochondrial distribution and shape after 72 h of irradiation were also evident using electron microscopy imaging (Figure 4). Irradiated cells showed mitochondria with unusually elongated profiles, similar to giant mitochondria. We additionally observed some small mitochondria in the cells after irradiation with blue light. In untreated cells, most of the mitochondria formed a dense cluster on one side of the nucleus. After blue light exposure, mitochondria were found in the entire perinuclear region.

### Expression of stress-related proteins

**Figure 5:**
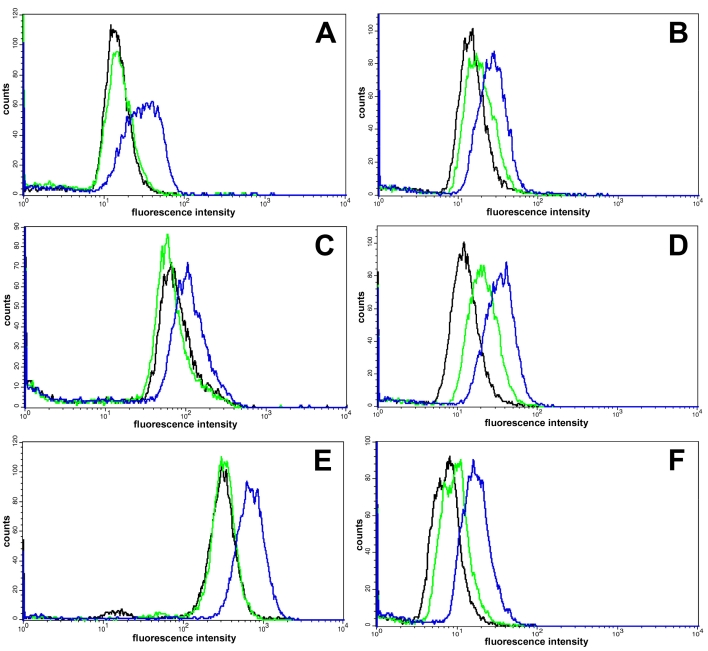
Effect of blue light on expression of stress-related proteins. The figure shows flow cytometric analysis of HO-1 (**A**), SOD-Mn (**B**), osteopontin (**C**), Cathepsin D (**D**), Hsp-27 (**E**), and CML (**F**) expression in ARPE-19 cells. Cells were irradiated at 1 mW/cm^2^ (blue) and 0.3 mW/cm^2^ (green) or unirradiated (black) for 24 h (**C, F**), 48 h (Hsp-27), or 72 h (**A, B, D**). Fluorescence intensity distribution correspond to expression of appropriate protein or CML expression. Blue light with an output power of 1 mW/cm^2^ enhanced expression of stress-related proteins.The expression of cathepsin D increased in cells also after a lower blue light output power of 0.3 mW/cm^2^. Results are representative of 3 separate experiments.

Flow cytometric analysis revealed blue-light-induced changes of stress-related cellular proteins (Figure 5, Table 1). The expression of HO-1, osteopontin, Hsp-27, SOD-Mn, and cathepsin D were examined in ARPE-19 cells following different exposure times to blue light. Blue light with an output power of 1 mW/cm^2^ (blue diagrams) enhanced HO-1, SOD-Mn, and cathepsin D expression in ARPE-19 cells after 72 h irradiation. Expression of of the anti-apoptotic glycoprotein osteopontin was enhanced after only 24 h and Hsp-27 after 48 h. With a lower output power of 0.3 mW/cm^2^, the stress reaction was diminished. Only cathepsin D expression increased in ARPE-19 cells after irradiation at 0.3 mW/cm^2^ (green diagrams).

**Table 1:** Quantitative analysis of flow cytometric results in Figure 5.

** ** **Stress-related protein**	**Blue light**	
	**0.3 mW/cm^2^**	**1 mW/cm^2^**
HO-1	1.2±0.2	4.3±0.6 *
SOD-Mn	1.3±0.1	4.2±0.5 *
Osteopontin	0.9±0.2	1.2±0.2 *
Cathepsin D	1.9±0.3*	5.3±0.8 *
Hsp-27	1.0±0.1	4.5±0.5 *
CML	1.2±0.1	3.7±0.4 *

Mean fluorescence intensities in blue light-irradiated cells relative to control cells. Results are representative of 3 separate experiments, each performed in triplicate. Mean fluorescence intensities in blue-light-irradiated cells relative to control cells. Asterisk (*) denotes p<0.05 versus control.

### Induction of CML-modified proteins

The blue-light-induced formation of intracellular CML-modified proteins in ARPE-19 cells was studied. After 24 h of exposure to blue light with an output power of 1 mW/cm^2^, cytofluorometrical analysis revealed an accumulation of the AGE product CML in ARPE-19 (Figure 5, Table 1).

### Cell cycle analysis

**Figure 6:**
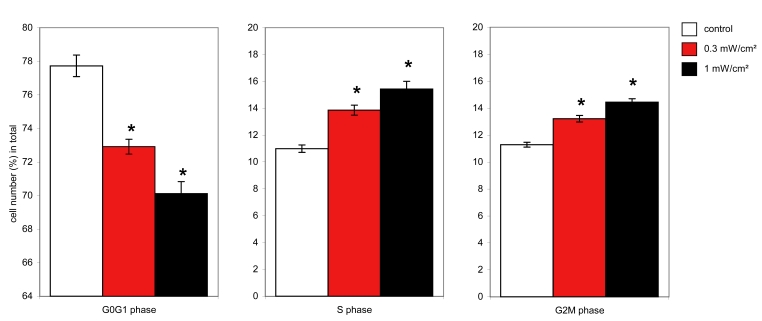
Effect of blue light on cell cycle. Flow cytometric analysis of cell cycle of the ARPE-19 cells in control cells (white bars) versus cells irradiated at 0.3 mW/cm^2^ (red bars) and 1 mW/cm^2^ (black bars) for 72 h. Diagrams show statistics of 5 separate experiments, each performed in triplicate. The differences between control cells and irradiated cells are significant; the asterisk (*) indicates a p<0.05 (one-way ANOVA and Bonferroni test).

We performed cell cycle analysis on cells irradiated with blue light for different time periods. Blue light treatment resulted in a relative increase in the G2M and S phase of the cell cycle in a dose-dependent manner. The maximum effect was observed at 72 h and did not increase with longer durations of irradiation (Figure 6). The population of cells in the G2M phase of the cell cycle reached 14.3% after 72 h of irradiation, while the control cells were at 11.3%. The percentage of cells in S phase was also higher in the irradiated cells (15%) compared to control cells (10.9%). Subsequently, the G0G1 phase of cell cycle decreased from 77.7% in control cells to 70.5% after blue light exposure.

## Discussion

Here we develop and characterize an in vitro model system in which blue light irradiation of RPE cells induces mild stress without causing cell death. Although most studies investigating the relationship between blue light, RPE cells, and AMD focused on the elucidating mechanisms of blue light-induced apoptosis [12,15,26-31], widespread RPE cell death is not generally seen in early AMD. In fact, epidemiologic data has demonstrated that RPE cell death, termed geographic atrophy, occurs in only 10%–15% of AMD patients and only at the latter stages of the disease [32 [32].

Using our model, we show that blue-light-illuminated RPE cells exhibit a stress response to low doses of stimuli that do not otherwise affect cell viability. Using a resazurin assay, we found decreased metabolic activity in ARPE-19 cells treated with blue light for 24 h. Viability was not affected as shown by trypan blue exclusion, propidium iodide staining, and apoptosis assays. Thus, changes in resazurin reduction must be due to a decrease in metabolic activity and not reduced viability. Upregulation of stress proteins is a known protective response to oxidative stress [33-39]. FACS analysis of cell stress-related proteins showed increased HO-1, osteopontin, Hsp-27, SOD-Mn, and cathepsin D expression after blue light treatment of ARPE-19 cells. These results confirm our hypothesis that stress signaling is part of the RPE reaction to blue light at low doses.

Blue light exposure in our system caused significant ROS production in ARPE-19 cells. These findings are consistent with other studies that demonstrated blue light could trigger intracellular ROS production [14,40,41]. There is evidence that blue light might interact with mitochondrial cytochrome and flavin oxidases to elevate ROS [42,43]. In vivo, this might lead to mitochondrial impairement and cell dysfunction over time [44]. In AMD, oxidative stress induced by light may be a key factor in RPE degeneration [2,8,26,28]. In our model system, intracellular elevated ROS production caused by blue light was not sufficient for initiation of apoptosis. This was expected, as RPE cells are able to tolerate oxidative stress without initiating cell death [7,45]. Stress signaling may explain our observation that blue light exposure leads to mitochondrial membrane depolarization after 24 h. A decrease in MMP is found in many apoptotic systems [46]. A lower MMP can result in a decreased energy metabolism in mitochondria. This indicates that blue light irradiation affects mitochondrial activity and leads to a lower of metabolic state. Irradiation for longer periods resulted neither in cell death nor in increased metabolic affection.

We propose that RPE cells are able to adapt to low doses of blue light stimuli. This was confirmed by our observation that mitochondrial shape and distribution vary after 72 h of blue light treatment as visualized by electron microscopy and JC1-staining. Formation of so-called mega mitochondria is described in the literature as a response to cell stress [47,48]. The elongation of mitochondria that we observed after 72 h may reflect the formation of mega mitochondria as a reaction to blue light. This likely represents an adaptation mechanism to the applied stress, thereby causing better resistance to external damage. The transfer of energy accumulated as the difference in proton electrochemical potentials within one small mitochondrion is inefficient in contrast to giant mitochondria. These structures are capable of energy transfer along extensive mitochondrial membranes, providing higher levels of respiration and energy turnover [49].

DNA cycle analysis revealed an increase in both S and G2M phases and therefore a decrease in the G_0_G_1_ phase in blue-light-irradiated ARPE-19 cells. The obtained values remained stable for more than 72 h of treatment. A complete cell cycle arrest would have resulted in a constant increase of cells entering the cell cycle. Here, blue light irradiation caused a temporary delay in cell cycle completion, probably at the last checkpoint [50-52]. In our case it is likely that after damage, repair, and adaptation to blue light, cells finally complete the cell cycle. This assumption is confirmed by the absence of differences in cell number between treated and untreated groups.

Strikingly, we found that blue light irradiation resulted in the formation of AGE products, particularly CML-modified proteins in ARPE-19 cells. CML modification is one of the most abundant AGEs formed under oxidative conditions [53-55]. Increased AGE levels are also found in skin elastin and collagen during UV-induced photoaging [56-58]. Reactive oxygen intermediates are involved in the formation of early glycation adducts such as CML [55,59]. Increasing published evidence suggests that the accumulation of AGEs in the retina could play a significant role in initiation and pathogenesis of AMD [60,61]. Cumulative prolonged blue light damage at the RPE level may contribute to the development of specific changes characteristic for early AMD, including RPE pigmentary abnormalities and formation of sub-RPE extracellular deposits [62,63].

Our in vitro-model system provides a simple and useful means to investigate the effects of phototoxicity in the RPE. Phototoxicity includes light-induced apoptosis at high irradiation intensities, but also the effect of blue light at nonapoptotic levels on human RPE cells. We demonstrated that RPE cells in vitro first react with stress signaling at low doses of stimuli. Cells adapt to the light-induced stress and therefore survive. Adaption over the long time includes an altered mitochondrial profile as well as cell cycle delay. It remains to be elucidated whether these changes in RPE cells alone lead to impaired visual functions in vivo. Future investigation will determine which pathways activate the adaptation process in response to blue light exposure.
